# Supplementary material for: Sustained-input switches for transcription factors and microRNAs are central building blocks of eukaryotic gene circuits
Source: Genome Biol. 2013 Aug 23;14(8):R85. doi: 10.1186/gb-2013-14-8-r85 (PMC4054853; doi:10.1186/gb-2013-14-8-r85)
Supplement: Additional file 5 — HTML Browsable Motif Output. Zipped folder containing all WaRSwap and FANMOD motif output, viewable in a web browser. [file gb-2013-14-8-r85-S5.ZIP › HTML_browsable_motif_output/WaRSwap_ath_tair9/sigs_WSWRSLDraw_2-2000.pvals.heatmaps.html/motif_id_46_000101110_tftype_ath_upstream_-2000_0.html]

```
BG_MODEL = Rnew2
MOTIF_ID = 46_000101110
TF_TYPE = ath
UPSTREAM = -2000_0


PVals
FN_0.2	FN_0.4	FN_0.6	FN_0.8
dg_60.genes	0.167	0	0.028	0.425
dg_70.genes	0.177	0	0.03	0.417
dg_80.genes	0.175	0	0.028	0.422

ZScores
FN_0.2	FN_0.4	FN_0.6	FN_0.8
dg_60.genes	0.985	2.653	1.911	-0.68
dg_70.genes	0.983	2.73	1.964	-0.662
dg_80.genes	1.008	2.629	1.94	-0.683

StDevs
FN_0.2	FN_0.4	FN_0.6	FN_0.8
dg_60.genes	137.928	65.817	22.089	1.158
dg_70.genes	136.723	63.871	21.47	1.166
dg_80.genes	134.621	66.552	22.317	1.123
```
